# 色素上皮衍生因子及其多肽对非小细胞肺癌增殖、凋亡及迁移的影响

**DOI:** 10.3779/j.issn.1009-3419.2021.102.43

**Published:** 2021-12-20

**Authors:** 志祥 晁, 西淳 秦, 才力 贾, 昊 秦, 昊 张

**Affiliations:** 1 221006 徐州，徐州医科大学附属医院胸外科 Department of Thoracic Surgery, Affiliated Hospital of Xuzhou Medical University, Xuzhou 221006, China; 2 221006 徐州，徐州医科大学第一临床医学院胸外科实验室 Thoracic Surgery Laboratory, The First College of Clinical Medicine, Xuzhou Medical University, Xuzhou 221006, China

**Keywords:** 色素上皮衍生因子, 肺肿瘤, 多肽, A549细胞, H1299细胞, 迁移, 凋亡, Pigment epithelium-derived factor, Lung neoplasms, Peptides, A549 cells, H1299 cells, Migration, Apoptosis

## Abstract

**背景与目的:**

色素上皮衍生因子（pigment epithelium-derived factor, PEDF）的抗肿瘤作用已被大量证实，然而其多肽的抗肿瘤作用报道较少，本研究旨在探讨PEDF及其多肽对非小细胞肺癌（non-small cell lung cancer, NSCLC）凋亡及迁移的影响。

**方法:**

选取肺癌A549细胞和H1299细胞为研究对象，通过给予细胞相同浓度的不同药物作为观察对象，分为正常组、PEDF处理组、34肽处理组、44肽处理组及34+44组合肽处理组。CCK-8法检测各组细胞的增殖活性；划痕实验检测细胞的迁移能力；免疫印迹法检测凋亡相关蛋白表达水平，如蛋白激酶3（RIP3）和半胱天冬酶3剪切体（cleaved-caspase-3）以及各组上皮-间充质转化（epithelial mesenchymal transition, EMT）标志物的表达水平，如钙黏蛋白（E-cadherin）和*α*-平滑肌肌动蛋白（*α*-SMA）；流式细胞术检测各组细胞凋亡率。

**结果:**

CCK-8结果显示PEDF及其各多肽分组均可抑制细胞的增殖其中34+44组合肽抑制效果最强（*P* < 0.05）；镜下观察发现PEDF及其多肽对A549细胞及H1299细胞均可起到抑制增殖及间充质转化的作用，且34+44肽组的抑制效果最明显；免疫印迹结果表明，与其他组别相比，34+44肽组细胞内Cleaved-Caspase-3及RIP3的表达量明显增高（*P* < 0.05），EMT蛋白E-cadherin的表达升高，*α*-SMA的表达量降低（*P* < 0.05）；流式细胞术结果表明，34+44组合肽组细胞的凋亡率明显高于对其他组（*P* < 0.05）；划痕实验表明，与所有组别相比，34+44肽组的愈合率最低（*P* < 0.05）。

**结论:**

34+44组合肽可以更好地促进NSCLC的凋亡，抑制NSCLC的迁移，从而抑制NSCLC的增殖。

## Abstract (en)

**Background and objective:**

The anti-tumor effect of pigment epithelium-derived factor (PEDF) has been widely confirmed. However, the anti-tumor effect of its peptides is rarely reported. This study aims to investigate the effects of PEDF and its peptides on the apoptosis and migration of non-small cell lung cancer (NSCLC).

**Methods:**

In this study, A549 cells and H1299 cells were selected as the research object, and the cells were divided into normal group, PEDF treatment group, 34 peptide treatment group, 44 peptide treatment group and 34+44 peptide treatment group by administering different drugs at the same concentration to the cells. The proliferation activity of cells in each group was detected by CCK-8 method; the migration ability of cells was detected by scratch test; the expression levels of apoptosis related proteins such as protein kinase 3 (RIP3) and cleaved-caspase-3 were detected by Western blot; the expression levels of epithelial mesenchymal transition (EMT) markers in each group, such as cadherin (E-cadherin) and *α*-smooth muscle actin (*α*-SMA) were detected by Western blot; the apoptosis rate of each group was detected by flow cytometry.

**Results:**

The results of CCK-8 showed that PEDF and its peptides could inhibit cell proliferation, and the inhibitory effect of 34+44 peptide was the strongest (*P* < 0.05); Observation under the microscope found that PEDF and its peptides can inhibit the proliferation and mesenchymal transformation of A549 cells and H1299 cells, and the inhibitory effect of the 34+44 peptide group is the most obvious; Western blot indicated that compared with other groups, the expressions of cleaved-caspase-3 and RIP3 in 34+44 peptide group were significantly higher (*P* < 0.05), and the expressions of EMT protein E-cadherin were higher, the expression of *α*-SMA decreased (*P* < 0.05); The results of flow cytometry showed that the apoptosis rate of 34+44 peptide group was significantly higher than those of other groups (*P* < 0.05); The scratch test showed that compared with all the other groups, the healing rate of 34+44 peptide group was the lowest (*P* < 0.05).

**Conclusion:**

34+44 combination peptide can better promote the apoptosis of NSCLC, inhibit the migration of NSCLC, and thereby inhibit the growth of NSCLC.

目前肺癌是世界上发病率和死亡率极高的恶性肿瘤^[1]^，据统计，非小细胞肺癌（non-small cell lung cancer, NSCLC）约占报告病例的85%^[2]^，近80%的NSCLC患者被诊断为晚期，晚期NSCLC患者的预后极差^[3]^。近年来，血管生成在肿瘤发展，生长和转移中的作用一直是研究的热点^[4]^。色素上皮衍生因子（pigment epithelium-derived factor, PEDF）是一种50 kDa分泌性的糖蛋白，具有多种生物学功能，包括抗血管生成、抗炎和神经营养^[5]^。越来越多的证据^[6]^表明，PEDF对多种类型肿瘤细胞具有抑制作用，如卵巢癌、胰腺癌、骨肉瘤等。然而，由于PEDF本身分子量较大，半衰期短，免疫原性较强，难以实现临床转化应用。多肽在多种疾病的治疗上具有特异性和有效性，并具有良好的药理动力学和较低的生产成本^[7]^。已在人类PEDF中鉴定出两个功能性基序，分别是34聚体（氨基酸位置Asp44-Asn77）和44聚体（氨基酸位置Val78-Thr121），它们分别负责抗血管生成活性和神经营养活性^[8]^。已有研究^[9]^证明PEDF能有效抑制肺癌的增殖和迁移，然而PEDF多肽的抗肿瘤作用鲜有报道。因此，探究短功能PEDF肽的抗肿瘤生物学活性具有潜在价值。本研究旨在探讨PEDF及其衍生多肽对肺癌细胞系A549细胞增殖、凋亡及迁移的影响，从而为肺癌的治疗提供新的思路。

## 1 材料与方法

### 1.1 细胞培养与试剂

A549细胞和H1299细胞购于上海中科院细胞库，A549细胞用含10%血清的高糖DMEM培养基，H1299细胞用含10%血清的高糖RPMI-1640培养基，在37 ℃、5%CO_2_的培养箱内常规培养，细胞生长汇合度达到90%以上时，消化传代培养。A549细胞和H1299细胞铺满培养皿60%左右时，分别对细胞进行药物处理，分为正常组、PEDF组（60 nmol/L）、34肽组（60 nmol/L）、44肽组（60 nmol/L）及34+44组合肽（60 nmol/L）；DMEM高糖培养基（美国HyClone公司）；RPMI-1640培养基（美国HyClone公司）；胎牛血清（四季青公司）；人重组PEDF蛋白34肽（氨基酸序列：DPFFKVPVNKLAAAVSNFGYDLYRVRSSTSPTTN）、44肽（氨基酸序列：PLSVATALSALSLGAEQRTESIIHRALYYDLISSPDIHGT）（生工生物工程上海股份有限公司）溶于无菌PBS中；CCK-8试剂盒（南京凯基公司）；Annexin V-FITC/PI凋亡检测试剂盒（南京凯基公司）。

### 1.2 CCK-8法

取对数生长期的细胞制成单细胞悬液，分别调整细胞密度为5×10^4^/mL，铺96孔板，每孔100 μL。细胞贴壁后，A549细胞系中，实验组各孔加入相同浓度的体积为3 μL的分组药物稀释的DMEM培养基，对照组加入等体积的培养基，每组设5个复孔，作用48 h后，各孔加入CCK-8试剂10 μL，置于37 ℃、5%CO_2_培养箱继续培养，450 nm波长处检测吸光度（optical density, OD）值。H1299细胞系中，实验组各孔加入相同浓度的体积为3 μL的分组药物稀释的RPMI-1640培养基，对照组加入等体积的培养基，每组设5个复孔，作用48 h后，各孔加入CCK-8试剂10 μL，置于37 ℃、5%CO_2_培养箱继续培养，450 nm波长处检测吸光度值。计算各组细胞生长的抑制率，抑制率（%）=[1-（对照组OD值-实验组OD值）/空白组OD值]×100%，实验重复3次。

### 1.3 形态学实验

取对数生长的细胞铺板于6孔板中，在细胞生长至约50%密度时，对不同分组加入药物处理，24 h后在光镜下观察各组细胞形态的变化。

### 1.4 免疫印迹法

细胞加入裂解液后经离心提取蛋白，使用BCA法测定蛋白浓度。配制聚丙烯酰胺凝胶，每孔按照20 μL体积上样，接下来依次进行电泳和转膜。转膜完成后使用5%脱脂奶粉室温封闭2 h。一抗1:1, 000稀释后4 ℃过夜孵育。根据一抗来源的不同选择二抗，稀释倍数为1:20, 000，避光室温条件下孵育2 h，采用Odyssey系统进行扫描。使用Image J对条带进行定量分析，各组目的蛋白灰度值与内参（β-tubulin）灰度值比值进行统计学分析。

### 1.5 划痕实验

划痕实验检测细胞的迁移能力，将A549细胞和H1299细胞以5×10^5^/mL的密度铺于6孔板内，在A549细胞系中加入含10%胎牛血清的DMEM培养基，在H1299细胞系中加入RPMI-1640培养基培养48 h，使之形成单层细胞。用200 μL移液枪枪头在单层细胞上纵横划痕。划痕操作后，将其分为5组，分别为空白对照组、PEDF组、PEDF-34肽组、PEDF-44肽组和PEDF-34+44组合肽组，分别于划痕之后的0 h、24 h在倒置荧光显微镜下观察（×40倍）并拍照。使用Image J对划痕愈合率进行分析。划痕愈合率（%）=（0 h划痕面积-24 h划痕面积）/0 h划痕面积×100%。

### 1.6 流式细胞术

流式细胞仪检测PEDF全蛋白，不同肽段（PEDF-34肽、PEDF-44肽）对A549细胞和H1299细胞凋亡的影响，取对数生长期的A549细胞和H1299细胞（2×10^5^/mL）接种于6孔板，培养24 h后弃去孔内的培养基，实验组分别加入相同浓度PEDF全蛋白、PEDF-34肽、PEDF-44肽及PEDF-34+44肽（浓度为20 mg/μL）稀释的培养基，对照组加入等体积培养基，置于37 ℃、5%CO_2_培养箱培养，加药48 h后，用不含EDTA的胰酶和预冷的PBS消化、离心、洗涤、收集细胞（按说明书操作，在流式细胞仪上检测凋亡），加入500 μL Binding Buffer重悬细胞，5 μL Annexin-V-FIT避光孵育15 min，加入5 μL PI混匀后避光5 min，流式细胞仪分析细胞凋亡率，实验重复3次。

### 1.7 统计学分析

计量资料以均数±标准差（Mean±SD）表示，采用SPSS 25.0统计软件进行统计学分析。多组间差异比较采用单因素方差分析，两组间比较采用*t*检验，检验水准α=0.05，以*P* < 0.05为差异有统计学意义。

## 2 结果

### 2.1 PEDF全蛋白、34肽、44肽及34+44组合肽对A549细胞和H1299细胞增殖的影响

CCK-8法检测各组OD值，在A549细胞系中，各实验组与对照组抑制率相比，抑制率最明显的为PEDF-34+44肽组55.91%±2.24%，44肽组为43.15%±3.43%、34肽组为33.02%±2.24%、PEDF组为26.07%±3.16%（图 1A、图 1B）。在H1299细胞系中，各实验组与对照组抑制率相比，抑制率最明显的为34+44肽组58.03%±2.99%，44肽组为44.45%±2.70%、34肽组为33.30%±3.08%、PEDF组为27.78%±2.53%，差异均有统计学意义（*P* < 0.05）（图 1C、图 1D）。上述结果表明，PEDF全蛋白及34肽、44肽对NSCLC A549细胞和H1299细胞的增殖具有抑制作用，34+44肽组抑制效果最为明显。

**图 1:**
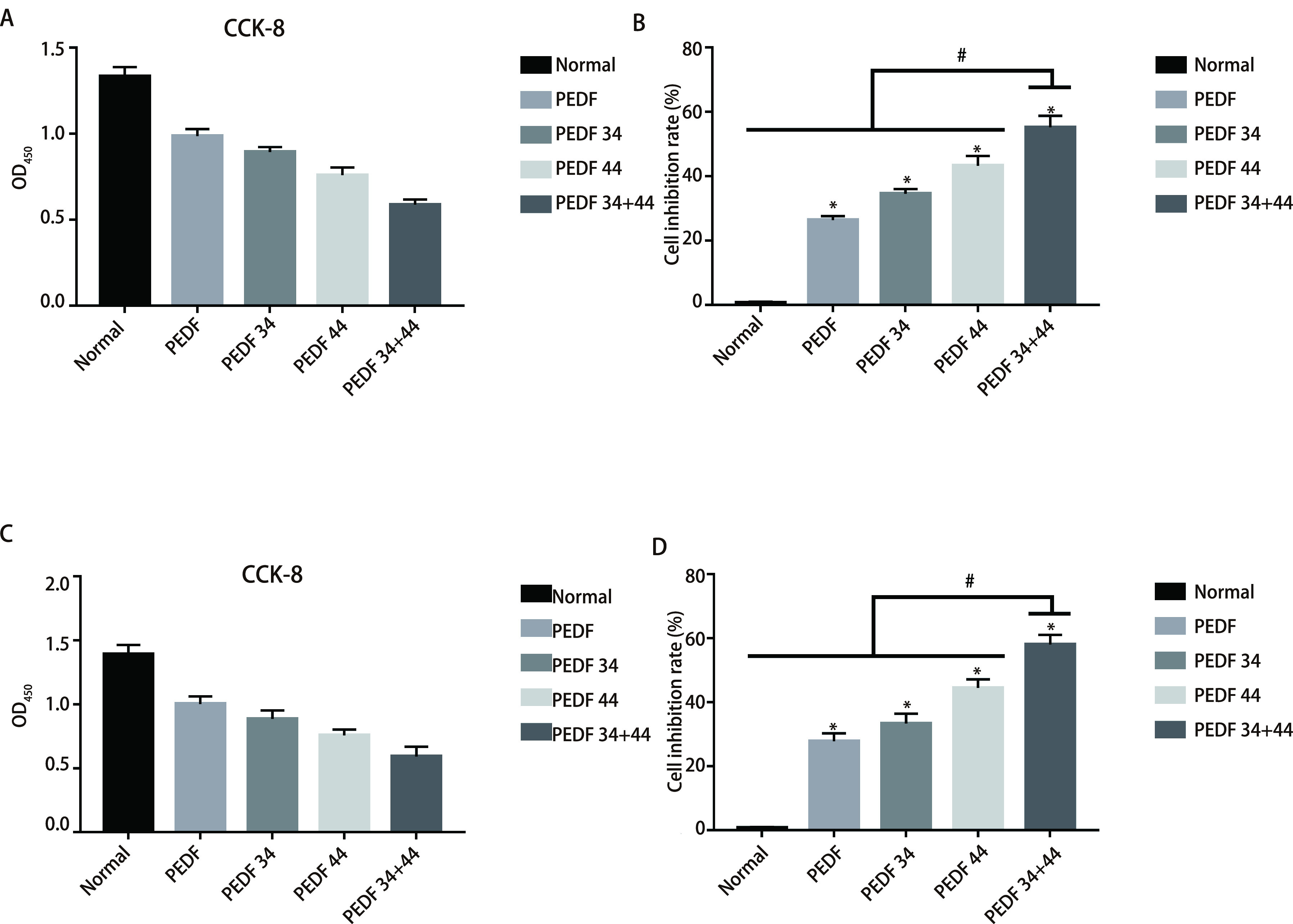
CCK8检测各组细胞增殖活性。A：A549细胞系中各组细胞的OD值；B：各组细胞增殖的抑制率及其相互比较。C：H1299细胞系中各组细胞的OD值；D：各组细胞增殖的抑制率及其相互比较。 CCK8 detects cell proliferation activity in each group. A: the OD value of each group of cells in the A549 cell line; B: the inhibition rates of cell proliferation in each group and their comparison with each other; C: the OD value of each group of cells in the H1299 cell line; D: the inhibition rate of cell proliferation in each group and their comparison with each other. ^*^ means that all groups have statistical differences compared with the normal group (*P* < 0.05); ^#^ means that all groups have statistical differences compared with the 34+44 peptide group (*P* < 0.05).

### 2.2 PEDF全蛋白、34肽、44肽及34+44组合肽对A549细胞和H1299细胞形态的影响

在加药24 h后，荧光显微镜观察各组细胞形态的变化，在两个细胞系中，与正常组相比，34+44肽组的细胞形态从长梭形变为饱满圆润的细胞数更多，依次为33肽组、PEDF组、44肽组。与正常组相比，细胞密度由高到低依次为PEDF组、34肽组、44肽组、34+44肽组（图 2）。因此得出，PEDF及其多肽对A549细胞及H1299细胞均可起到抑制增殖及间充质转化的作用，且34+44肽组的抑制效果最明显。

**图 2:**
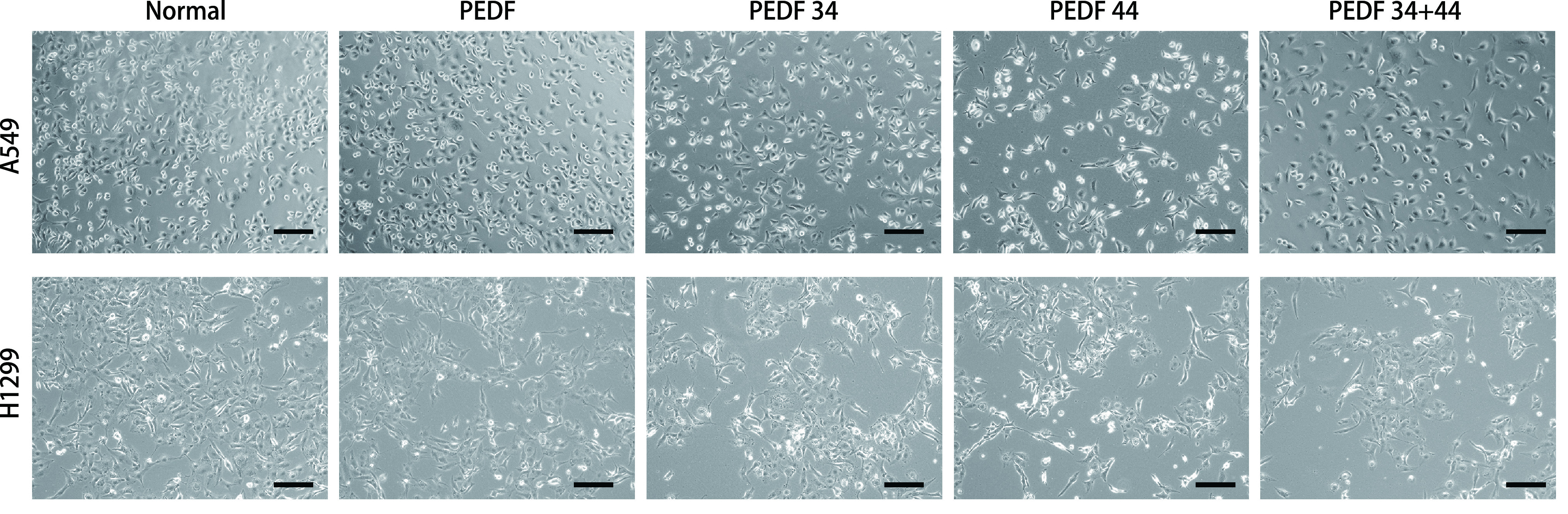
光学显微镜去观察药物处理24 h后各组细胞形态的变化（×200, bar=50 μm） Optical microscope to observe the changes of cell morphology in each group after 24 h of drug treatment (× 200, bar=50 μm)

### 2.3 PEDF全蛋白、34肽、44肽及34+44组合肽对A549细胞和H1299细胞凋亡的影响

蛋白免疫印迹结果显示：在两个细胞系中，凋亡相关蛋白Cleaved-Caspase-3及RIP3的表达量增加最为明显的组别，依次为34+44肽组、44肽组及34肽组，各实验组与正常组相比，差异均有统计学意义（*P* < 0.05）；各组与34+44肽组相比，差异均有统计学意义（*P* < 0.05）（图 3D-图 3F；图 4D-图 4F）；流式细胞术结果显示：在A549细胞系中，与对照组相比，细胞凋亡率最高的是34+44组合肽组为23.29%±2.36%；对照组为3.07%±0.21%；PEDF组为14.71%±0.78 %；34肽组为13.47%±1.24%以及44肽组为16.53%±0.88%。在H1299细胞系中，与对照组相比，细胞凋亡率最高的是34+44组合肽组为23.63%±2.40%；对照组为3.59%±0.29%；PEDF组为12.73%±2.35%；34肽组为12.51%±2.24%以及44肽组为14.48%±1.24%。可见不同组别与对照组相比，实验组细胞凋亡率均高于对照组（*P* < 0.05），且34+44肽组细胞凋亡率高于其他组（*P* < 0.05），并且由流式结果可以得出各实验组对细胞促凋亡作用主要表现为晚期凋亡（图 6）。因此得出：PEDF全蛋白及不同肽段（34肽、44肽）对A549细胞和H1299细胞的凋亡具有促进作用，34+44组合肽组促进凋亡效果最为明显。

**图 3:**
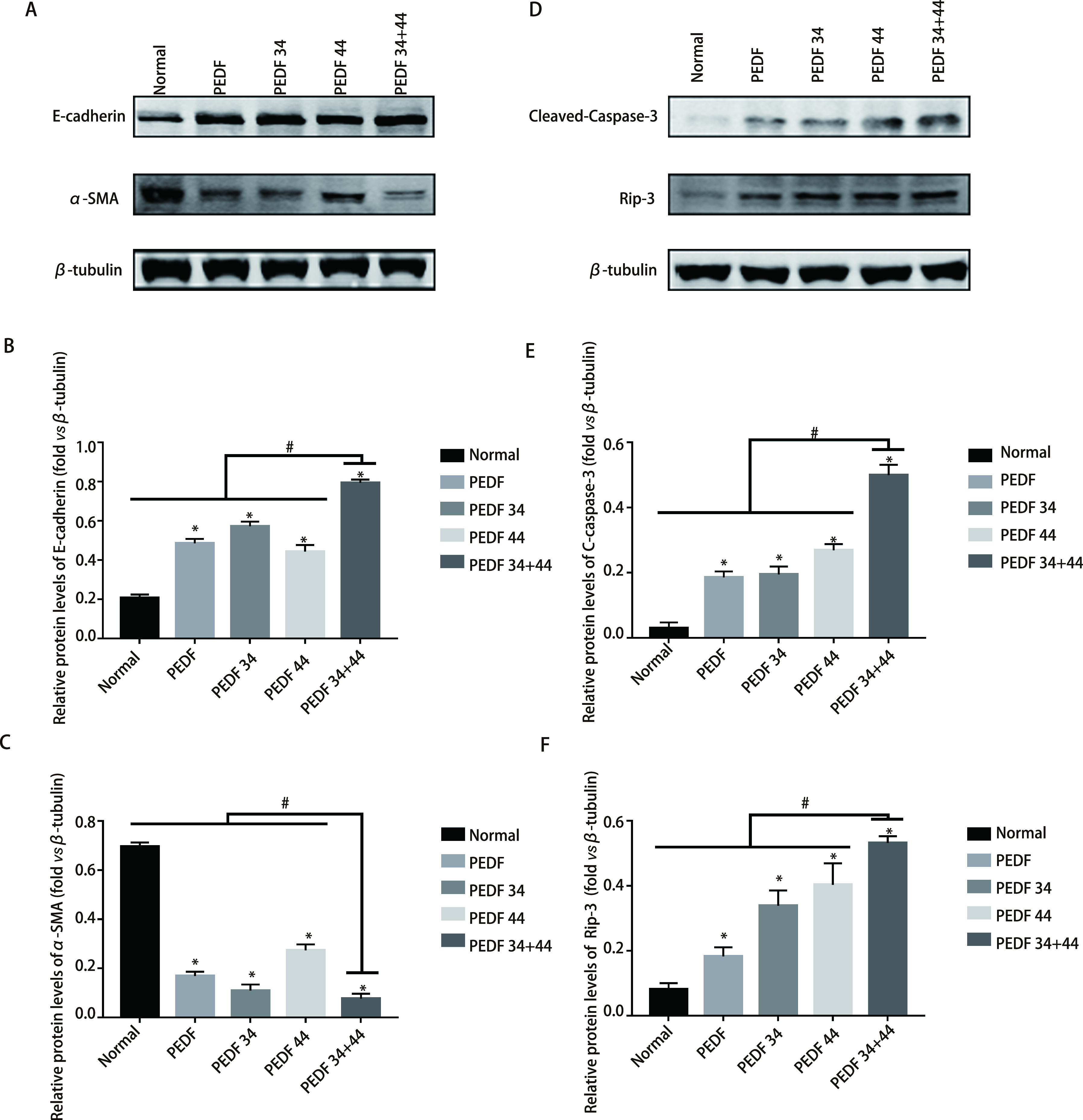
蛋白印迹检测各组EMT相关表达及凋亡相关蛋白表达情况。A：检测A549细胞系中迁移相关蛋白（E-cadherin、*α*-SMA）的表达量；B、C：相关蛋白表达量统计图；D：A549细胞系中凋亡相关蛋白（Clevead-Caspase-3、Rip-3）的表达情况；E、F：相关蛋白表达统计图。 Western blot was used to detect the expression of EMT-related and apoptosis-related proteins in each group. A: the detection of the expression of migration-related proteins (E-cadherin, *α*-SMA) in the A549 cell line; B and C: statistical diagrams of the expression of related proteins; D: the expression of apoptosis-related proteins (Clevead-Caspase-3, Rip-3) in the A549 cell line; E and F: statistical diagrams of related protein expression. ^*^ means that all groups have statistical differences compared with the normal group (*P* < 0.05); ^#^ means that all groups have statistical differences compared with the 34+44 peptide group (*P* < 0.05).

**图 4:**
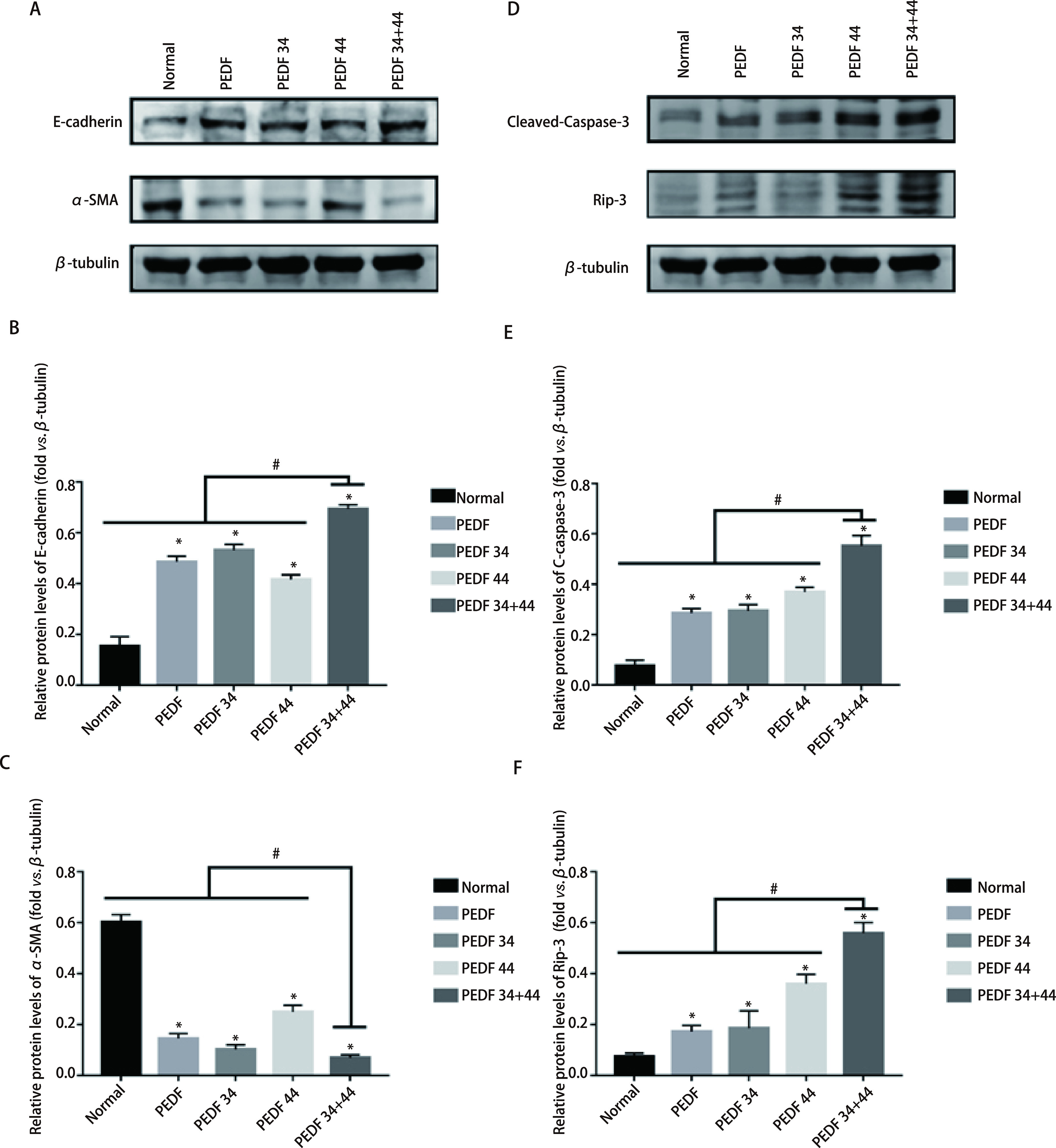
蛋白印迹检测各组EMT相关表达及凋亡相关蛋白表达情况。A：检测H1299细胞系中迁移相关蛋白（E-cadherin、*α*-SMA）的表达量；B、C：相关蛋白表达量统计图；D：H1299细胞系中凋亡相关蛋白（Clevead-Caspase-3、Rip-3）的表达情况；E、F：相关蛋白表达统计图。 Western blot was used to detect the expression of EMT-related and apoptosis-related proteins in each group. A: the detection of the expression of migration-related proteins (E-cadherin, *α*-SMA) in the H1299 cell line; B and C: statistical diagrams of the expression of related proteins; D: the expression of apoptosis-related proteins (Clevead-Caspase-3, Rip-3) in the H1299 cell line; E and F: statistical diagrams of related protein expression. ^*^ means that all groups have statistical differences compared with the normal group (*P* < 0.05); ^#^ means that all groups have statistical differences compared with the 34+44 peptide group (*P* < 0.05).

**图 5:**
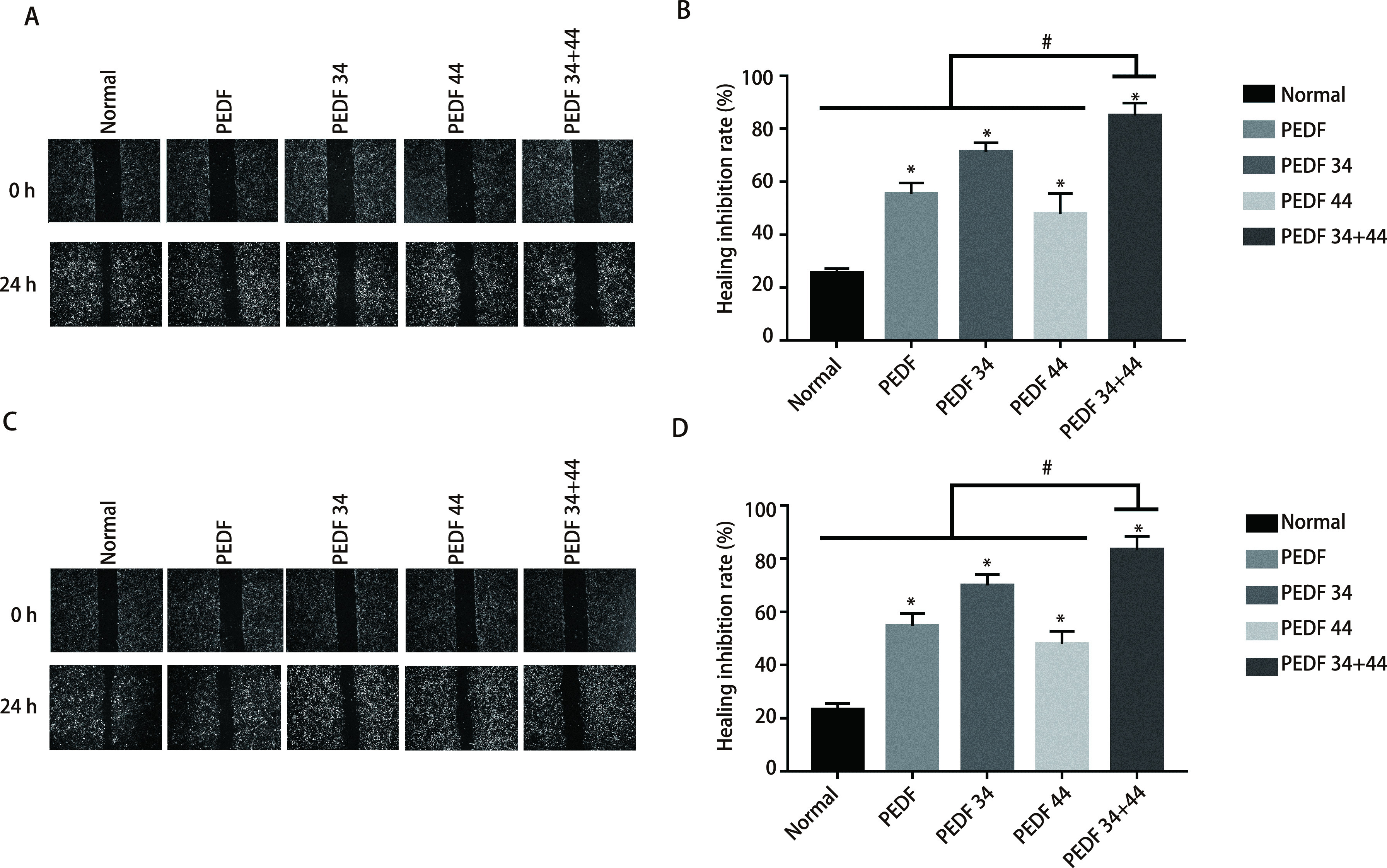
划痕实验检测各组细胞迁移能力的变化。A：A549细胞各组伤口愈合实验；B：愈合抑制率统计图；C：H1299细胞各组伤口愈合实验；D：愈合抑制率统计图。 Wound healing test to detect changes in cell migration ability of each group. A: the wound healing experiment of each group of A549 cells; B: the statistical graph of the healing inhibition rate; C: a wound healing experiment of each group of H1299 cells; D: a statistical graph of the healing inhibition rate. ^*^ means that all groups have statistical differences compared with the normal group (*P* < 0.05); ^#^ means that all groups have statistical differences compared with the 34+44 peptide group (*P* < 0.05).

**图 6:**
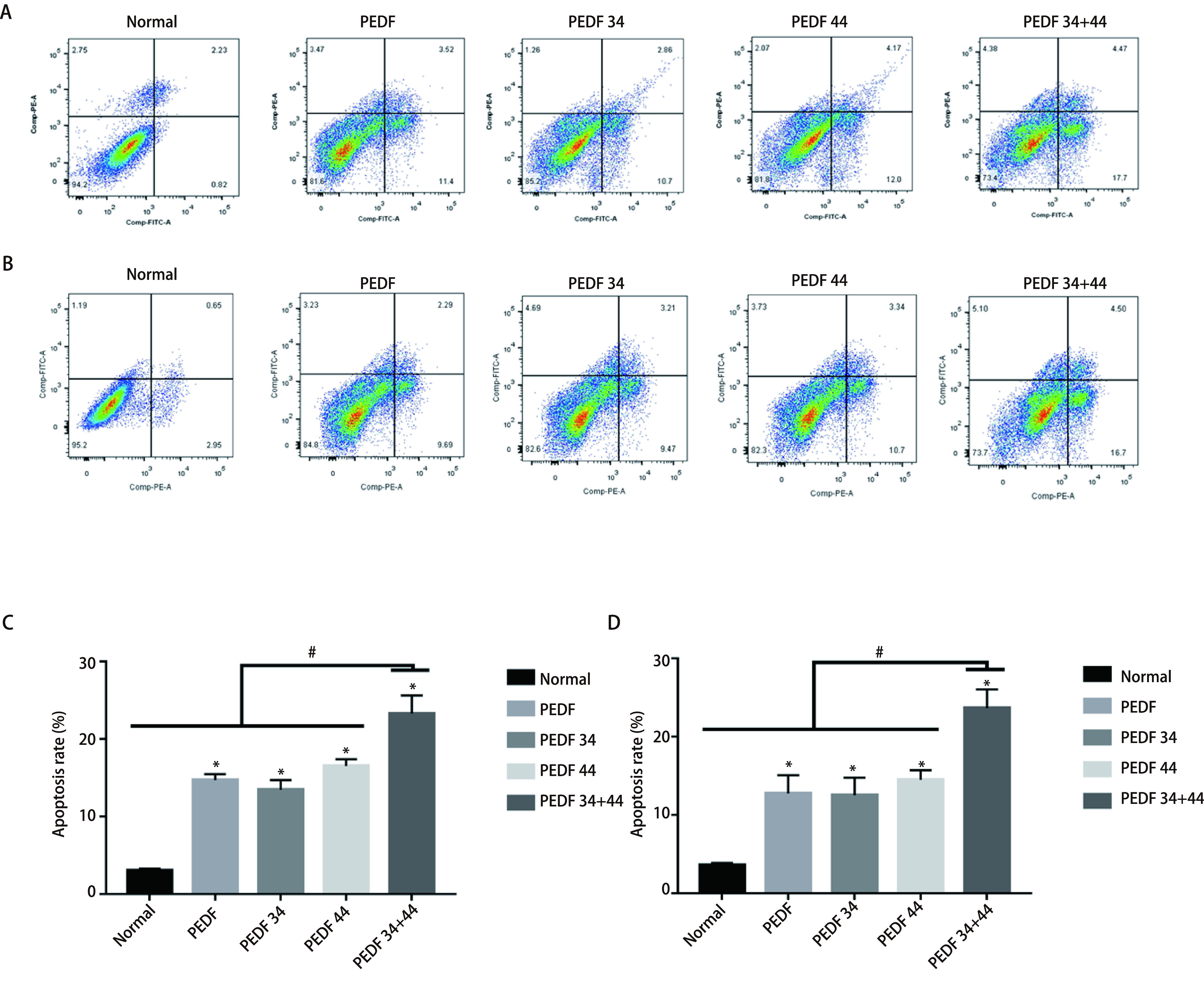
流式细胞术检测各组细胞的凋亡情况。A：A549细胞系中各组细胞凋亡情况；B：H1299细胞系中各组细胞凋亡情况；C、D：A549细胞系及H1299细胞系各组细胞凋亡率及其比较。 Flow cytometry to detect the apoptosis of each group of cells. A: shows the apoptosis of each group in the A549 cell line; B: the apoptosis of each group in the H1299 cell line; C and D: the apoptosis rate of each group of the A549 cell line and the H1299 cell line and their comparison. ^*^ means that all groups have statistical differences compared with the normal group (*P* < 0.05); ^#^ means that all groups have statistical differences.

### 2.4 PEDF全蛋白、34肽、44肽及34+44组合肽对A549细胞和H1299细胞的迁移能力影响

蛋白免疫印迹结果显示：在两个细胞系中，各组EMT相关蛋白表达，其中E-cadherin表达量依次为34+44肽组、44肽组、PEDF组，各实验组与正常组比较均有统计学差异（*P* < 0.05），各组与34+44肽组相比，差异均有统计学意义（*P* < 0.05）；*α*-SMA在34+44肽组的减少最为明显，依次为34肽组、PEDF组，各实验组与正常组比较均有统计学差异（*P* < 0.05），各组与34+44肽组相比，差异均有统计学意义（*P* < 0.05）（图 3A-图 3C；图 4A-图 4C）。这些结果表明34+44肽组抑制效果最好，其次为PEDF-34肽组。划痕实验结果显示，与所有组别相比，34+44肽组的愈合率最低，其他组别与正常组相比，愈合率均有差异，差异有统计学意义（*P* < 0.05）（图 5）。因此得出：PEDF全蛋白及不同肽段（PEDF-34肽、PEDF-44肽）对A549细胞和H1299细胞均有抑制迁移作用，PEDF-34+44组合肽组抑制效果最为明显。

## 3 讨论

目前，虽然在肺癌的诊断和治疗方面取得重大进展，但肿瘤增殖和转移仍是阻碍临床治疗效果的主要原因。PEDF由*SERPINF1*基因编码，是一种具有高度保守的折叠构象的分泌性多效单体糖蛋白，在机体多种组织中均有表达。PEDF最初被鉴定为视网膜色素上皮细胞中的神经营养因子，但现在PEDF被公认为最有效的内源性抗血管生成因子之一。

随着蛋白组学的发展，小分子多肽的研究越来越受关注，其保留全蛋白某种特异性功能，具有较高的生物利用率及生物安全性，同时还能避免全蛋白带来的免疫反应。已在PEDF中鉴定出两个功能性肽段，分别是34肽（氨基酸位置Asp44-Asn77）和44肽（Val78-Thr121）。有研究显示，34mer可诱导内皮细胞凋亡而抑制血管再生。44mer具有神经营养及细胞保护作用，但却不能抑制血管再生。随着对PEDF生物学功能研究的深入，PEDF已被证明可以抑制多种恶性肿瘤的发生发展，不仅能靶向肿瘤异常的微血管系统^[10, 11]^，还能直接抑制肿瘤细胞增殖或介导其凋亡^[12]^。然而，目前为止，关于PEDF衍生肽段的抗肿瘤特性的作用却知之甚少。

我们研究发现，在抑制A549细胞和H1299细胞增殖及促凋亡方面，两种PEDF衍生多肽效果均优于PEDF全蛋白，其中44肽效果最好。迁移实验结果显示出34肽对细胞有较强的抑制作用，但44肽效果不佳，WB的结果也证实这一点。值得注意的是，34+44组合肽在抑制增殖、迁移和促凋亡方面均表现最优。与全蛋白相比，34肽和44肽分子量更小，结构更为简单，可能更易于进入细胞内与相应受体结合，从而发挥特异性功能。由于多肽的氨基酸序列不同，因而会发挥不同的功能，但所选序列源自于同一蛋白，故而功能会有可能相似又不全相同。综合整个实验结果，这种组合用药方式能够有效抑制细胞的增殖和迁移，同时又能明显促进细胞的凋亡。

我们的研究尚有不足之处，本实验仅初步研究了34肽和44肽对NSCLC的生物学作用，作用机制尚不明确。PEDF存在多种受体，如层黏连蛋白受体（laminin receptor, LR）和脂肪甘油三酯脂肪酶（adipose triacylglyceride lipase, ATGL），这些受体或结合位点在很多细胞中表达，包括肿瘤细胞、内皮细胞、巨噬细胞、平滑肌细胞、骨骼肌细胞^[13]^。PEDF可与这些受体结合发挥生物学效应。除此之外，PEDF还具有胶原蛋白I和糖胺聚糖（包括肝素和透明质酸）的结合位点^[14]^。34-mer被认为是LR的结合位点，而44-mer则是PEDF与ATGL（也称PEDFR）结合的位点^[15]^。34肽和44肽不同的生物学作用，可能涉及不同的膜上受体或信号通路，这仍需要进一步研究。总之，34+44组合肽在抑制肺癌细胞增殖、迁移及促进细胞凋亡显现出良好的生物学效应，这种组合用药的方式不仅抗肿瘤效果显著，且能够极大降低生产成本，具有临床转化的可能，同时也为肺癌联合药物的研发提供一个策略。
